# MetaPhlAn 4 profiling of unknown species-level genome bins improves the characterization of diet-associated microbiome changes in mice

**DOI:** 10.1016/j.celrep.2023.112464

**Published:** 2023-05-03

**Authors:** Paolo Manghi, Aitor Blanco-Míguez, Serena Manara, Amir NabiNejad, Fabio Cumbo, Francesco Beghini, Federica Armanini, Davide Golzato, Kun D. Huang, Andrew M. Thomas, Gianmarco Piccinno, Michal Punčochář, Moreno Zolfo, Till R. Lesker, Marius Bredon, Julien Planchais, Jeremy Glodt, Mireia Valles-Colomer, Omry Koren, Edoardo Pasolli, Francesco Asnicar, Till Strowig, Harry Sokol, Nicola Segata

**Affiliations:** 1 Department CIBIO, University of Trento, Trento, Italy; 2 IEO, European Institute of Oncology IRCCS, Milan, Italy; 3 Department of Microbial Immune Regulation, Helmholtz Centre for Infection Research, Braunschweig, Germany; 4 Gastroenterology Department, Sorbonne Université, INSERM, Centre de Recherche Saint Antoine, CRSA, AP-HP, Saint Antoine Hospital, 75012 Paris, France; 5 Paris Centre for Microbiome Medicine (PaCeMM) FHU, Paris, France; 6 INRAE, UMR1319 Micalis & AgroParisTech, Jouy en Josas, France; 7 Azrieli Faculty of Medicine, Bar-Ilan University, Safed, Israel; 8 Department of Agricultural Sciences, University of Naples, Naples, Italy; 9 Centre for Individualised Infection Medicine (CiiM), a joint venture between the Helmholtz-Centre for Infection Research (HZI) and the Hannover Medical School (MHH), Hannover, Germany

**Keywords:** mouse microbiome, metagenomics, diet, uncharacterized microbial species, MetaPhlAn

## Abstract

Mouse models are key tools for investigating host-microbiome interactions. However, shotgun metagenomics can only profile a limited fraction of the mouse gut microbiome. Here, we employ a metagenomic profiling method, MetaPhlAn 4, which exploits a large catalog of metagenome-assembled genomes (including 22,718 metagenome-assembled genomes from mice) to improve the profiling of the mouse gut microbiome. We combine 622 samples from eight public datasets and an additional cohort of 97 mouse microbiomes, and we assess the potential of MetaPhlAn 4 to better identify diet-related changes in the host microbiome using a meta-analysis approach. We find multiple, strong, and reproducible diet-related microbial biomarkers, largely increasing those identifiable by other available methods relying only on reference information. The strongest drivers of the diet-induced changes are uncharacterized and previously undetected taxa, confirming the importance of adopting metagenomic methods integrating metagenomic assemblies for comprehensive profiling.

## Highlights

• The mouse gut microbiome is still dominated by unknown microbial taxa
• Considering unknown taxa improves diet-microbiome associations in mice
• The strongest biomarkers of different dietary fat intake are unknown taxa
• MetaPhlAn 4 can accurately profile unknown taxa in mice

## Graphical abstract

**Figure:**
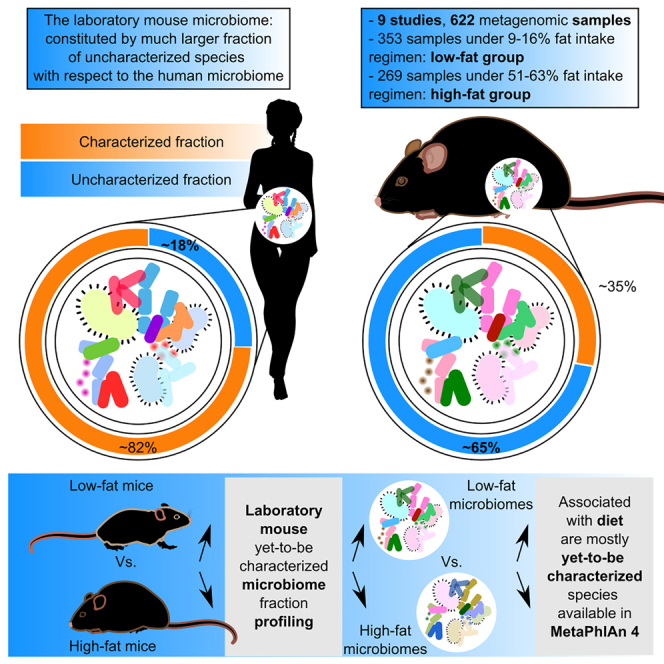

## Teaser

The mouse microbiome is still dominated by unknown taxa, and their definition is fundamental to understanding the diet-microbiome interplay in the mouse. Using MetaPhlAn 4, Manghi et al. show that this interplay can be accurately characterized by profiling the still-uncultivated fraction of the mouse microbiome.

## Introduction

Evolutionary, anatomical, and physiological proximity to humans make the mouse a successful model organism for biomedical research. Ease of breeding, validated disease models, and fast proliferation, as well as the possibility to perform multi-generation experiments and diet-related interventions, established mice as the main preclinical model for the study of the human gut microbiome.^1^^,^^2^^,^^3^^,^^4^^,^^5^^,^^6^ In mice, microbiome experiments can be conducted while controlling for several variables such as genetics, nutritional or pharmacological exposures, and other experiment-confounder factors.^1^^,^^2^^,^^3^^,^^5^^,^^6^^,^^7^^,^^8^^,^^9^^,^^10^^,^^11^^,^^12^^,^^13^^,^^14^^,^^15^^,^^16^^,^^17^^,^^18^^,^^19^ However, because the composition of the microbiome of laboratory and wild mice is different from that of humans,^3^^,^^4^ the mouse microbiome structure and diversity is far from being comprehensively addressed, with consequent limitations for fundamental and translational research in mice.

Analyses of the microbiome features characterizing diet, disease, and phenotype-related changes in mice have been extensively performed using 16S rRNA gene amplicon sequencing,^5^^,^^14^^,^^15^^,^^20^^,^^21^^,^^22^^,^^23^ which, despite the reduced costs, can be considered limited in its phylogenetic, taxonomic, and functional resolution. The high-resolution shotgun metagenomic approach, which is now the standard in human microbiome studies,^24^ is still much less employed in mouse studies for the lack of reference genomes covering the majority of the members of the mouse microbiome.^25^ Efforts at cataloging *de novo* the diversity of the mouse microbiome by systematic bioinformatic assembly of mice metagenomes have been undertaken,^25^^,^^26^^,^^27^^,^^28^^,^^29^ but it remains challenging to efficiently exploit them for new studies and in support of reference-based taxonomic profiling.

In this work, we show how the mouse gut microbiome and its links with nutritional patterns can be investigated accurately and comprehensively via shotgun metagenomics by leveraging a computational taxonomic profiling approach called MetaPhlAn 4,^30^ which integrates massive assemblies in its database. MetaPhlAn 4 considers over 22,718 metagenome-assembled genomes (MAGs) retrieved from 1,906 mouse-derived fecal, cecal, and ileal metagenomes with the species-level genome bin (SGB) strategy.^31^ By applying the approach on a large and heterogeneous catalog of mouse microbiomes, we show that accounting for metagenomically defined species is necessary in the context of nutritional studies in mouse models. We also found that the microbial species not detectable by mapping against genomes from isolates account for the greatest proportion of the diet-associated microbiome changes.

## Results

### A multi-cohort dataset for studying the impact of high-fat diets on the mouse gut microbiome

To study the influence of a high-fat diet on the mouse gut microbiome, we collected publicly available metagenomic datasets that assessed the mouse microbiome with respect to the content and the variable amount of fat in the diet (Table S1). For the collected samples, we manually curated and validated relevant mouse covariates including age, antibiotic usage, multiple time points when available, genetic background, sampling body-site, and dietary information (Table S2). On diet, we specifically focused on the percentage of calories derived from fat in the diet (from here on referred to as “fat percentage”) and on the time elapsed since the start of the diet intervention (Table S2). We initially retrieved 623 samples from 15 public datasets, which were reduced to 525 samples from eight datasets by retaining only datasets with at least two dietary treatments (either a high-fat and a low-fat group or multiple dietary fat-intake groups) and a minimum of 20 total samples. Additionally, we sequenced the cecal and ileal gut microbiomes of a mouse microbiome study in which mice were subjected to multiple types of gut microbiome perturbations, including several dietary regimens and antibiotic treatments, for a total of 97 metagenomes (see STAR Methods). Overall, we analyzed nine datasets and 622 samples (269 samples following a high-fat-intake regimen, average 57% fat content, 95% confidence interval (CI) of [0.51, 0.63]; and 353 following a low-fat-intake regimen, average 13%, 95% CI [0.09, 0.16]) (Tables S1 and S2).

### Reprofiling of the mouse gut microbiome evinces the dominant presence of uncharacterized microbial species

To perform accurate taxonomic profiling of the integrated cohorts for all species represented by available isolates and MAGs (average sequencing depth = 46.9 million, 95% CI [7.6, 86]), we applied MetaPhlAn 4.^30^ MetaPhlAn 4 uses the SGB approach^31^ to group both reference genomes and MAGs into known or unknown species that are then labeled kSGBs and uSGBs. An unknown species (uSGB) is thus a proxy for a microbial species that remains uncultivated and whose existence relies on information from metagenomic assembly. By detecting and quantifying both uSGBs and known SGBs (kSGBs), the approach enables profiling also the fraction of the microbiome that is not represented by existing reference genomes. Indeed, after incorporating 22,718 MAGs reconstructed from 1,906 mouse gut metagenomes from multiple sources (Table S3), MetaPhlAn 4 was able to identify and quantify 336 mouse-associated uSGBs that were not captured by the MAG-reconstructing procedure performed on the same samples, highlighting the strength of this approach. In the 622 samples considered in this study, we identified a total of 740 microbial SGBs (425 uSGBs and 316 kSGBs; average number of SGBs per sample 142, 95% CI [28.32, 255.68]) from 703 distinct species, greatly overcoming the previous detection of only 197 species by MetaPhlAn 3.

**Figure 1:**
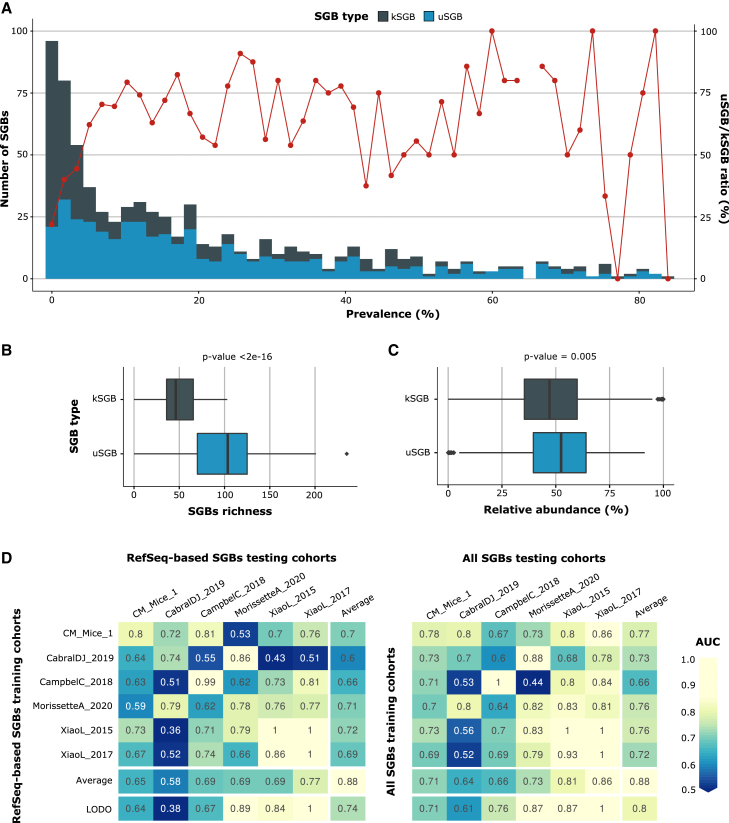
The mouse gut microbiome is dominated by uncharacterized microbial species that are highly relevant to improve the link between the microbiome and host dietary regimen (A) Number (barplot and left-side y axis) and ratio (line plot and right-side y axis) of k- and uSGBs for each 2% prevalence interval when considering all the 622 samples in the nine cohorts used in the paper. SGB prevalence is computed as the average prevalence across the datasets. The 2% bin size for the histogram has been chosen for visualization purposes. (B) Distribution of k- and uSGBs detected per sample. The box marks the distribution between the 25th and 75th percentiles, and whiskers are extended for 1.5× interquartile range (IQR). p value assessed via two-tailed Wilcoxon signed-rank test. (C) Distribution of relative abundance of k- and uSGBs per sample. p value assessed via two-tailed Wilcoxon signed-rank test. The box marks the distribution between the 25th and 75th percentiles, and whiskers are extended for 1.5× IQR. (D) Cross-prediction matrices for the prediction of a high-fat vs. a low-fat diet using a random forest classifier trained on arcsine-square-rooted relative abundances of SGBs spanned by RefSeq reference genomes (left) or all the SGBs available in MetaPhlAn 4 (right). Cells in the matrices represent AUCs obtained by the classifier trained on the corresponding row-dataset and tested on the corresponding column-dataset. Diagonal values are 10-fold cross-validations of AUC values. The “LODO” row reports the leave-one-dataset-out AUC values obtained by training the algorithm on each cohort but one and testing it on the left-out cohort, iteratively on all the cohorts. In the machine-learning experiments, we considered only samples not undertaking antibiotic treatments, and datasets with at least 20 samples from both types of diets.

We then evaluated single SGB prevalence, estimating the overall prevalence as the average of the prevalence across the nine datasets. We found the number of uSGBs with >50% prevalence in the considered cohort to be greater than the number of highly prevalent kSGBs (51 uSGBs vs. 25 kSGBs), and uSGBs statistically outnumbered kSGBs across all prevalence percentiles (p = 4.5 × 10^−4^, binomial test, Figure 1A). By considering the per-sample richness, we further found that the mouse gut microbiome harbors more uSGBs than kSGBs (average per-sample count = 95, 95% CI [10.72, 179.28], vs. 49, 95% CI [9.8, 88.2], respectively, Wilcoxon signed-rank test, p < 2 × 10^−16^; Figure 1B). uSGBs account for a higher relative abundance than kSGBs in individual microbiome samples (average per-sample 50.88%, 95% CI [46.95, 54.81] for uSGBs vs. 48.94%, 95% CI [45.01, 52.87] for kSGBs, Wilcoxon signed-rank test, p = 0.005; Figure 1C). Overall, these results support the need to also access and characterize the unknown fraction of the mouse gut microbiome.

### Accounting for uSGBs improves the discrimination between high- and low-fat-diet interventions

We then evaluated the impact of a high-fat diet on the microbiome of laboratory mice through changes in alpha- and beta-diversity. Shannon diversity increased in the high-fat groups of six datasets (average standardized mean difference [aSMD] = 1.16, 95% CI [1.06, 1.26], four significant at p < 0.05 [Wilcoxon signed rank]) compared with low-fat groups, but these alpha-diversity increases were not significant when confounders were taken into account (see STAR Methods and Table S2). Beta-diversity was analyzed in each dataset using the Permanova analysis of Bray-Curtis dissimilarity matrices and found significance in eight datasets out of nine (univariate Permanova p < 0.05; Figure S1A). Measuring the group mean differences between pairwise distances in low- and high-fat mice showed that six out of nine of the low-fat groups were more variable (overall aSMD = 0.71, 95% CI [0.004, 1.416]) than the corresponding high-fat groups. Permutation tests by multivariate Permanova correcting by study ID, mouse strain, age, sex, sampling body-site, and diet type (high-fat or low-fat) identified the study ID as the most important variable in determining the variability of the microbiome composition (adjusted R^2^ = 36%), followed by the mouse strain (adjusted R^2^ = 21%). Because the administration of a high-fat or low-fat diet accounted for a smaller percentage of the total microbiome variability (adjusted R^2^ = 3%) compared with the experimental differences reflected in study ID variability (Figure S1B and Table S4), we investigated diet-associated changes with cross-study and multivariate meta-analysis models that explicitly account for such study setting effects.

To further assess the association between the mouse gut microbiome and high- vs. low-fat diet regimen, we used a random forest^32^ classifier approach to link the dietary conditions with the presence and abundance of the SGBs present in the samples.^33^^,^^34^^,^^35^ We built two cross-prediction matrices^36^^,^^37^ by (1) training and evaluating the classifier in cross-validation (CV) on each dataset separately, (2) training a classifier on one dataset and applying it on a different dataset, and (3) training a classifier on all but one dataset and applying it on the left-out dataset (leave-one-dataset-out [LODO]).^36^^,^^37^^,^^38^^,^^39^ When considering only samples without antibiotic interventions (Table S2), the microbiome-based models achieved high discrimination between mice fed with high-fat vs. low-fat diets in all the prediction settings (Figure 1D), and substantial improvements were obtained when using the MetaPhlAn 4 profiles that incorporate all the SGBs compared with those obtained when only taxa present in RefSeq^40^ are included (RefSeq-based SGBs, see STAR Methods). While the performance in CV did not substantially change between the two CV settings, it markedly increased in the LODO setting when incorporating the MetaPhlAn 4 database, resulting in an average area under the curve (AUC) of 0.80 (compared with AUC 0.74 with RefSeq-based SGBs only) (Figure 1D). Similar improvements were achieved when considering samples from mice which undertook antibiotic treatments, reaching 0.92 average AUC in CV and 0.79 in LODO (compared with 0.91 and 0.72 average using only RefSeq-based SGB AUC, respectively; Figure S2). Overall, this suggests that species that are well characterized are sufficient to reach accurate intra-cohort predictions (CV), while the inter-cohort (LODO) performance that is a better proxy for model generalization appears to be heavily dependent on uSGBs. Incorporating MAGs from yet-to-be-characterized species can thus enable finding stronger reproducible associations with conditions of interest such as dietary regimes.

### Most cross-dataset microbial biomarkers of high- vs. low-fat diet are uSGBs

**Figure 2:**
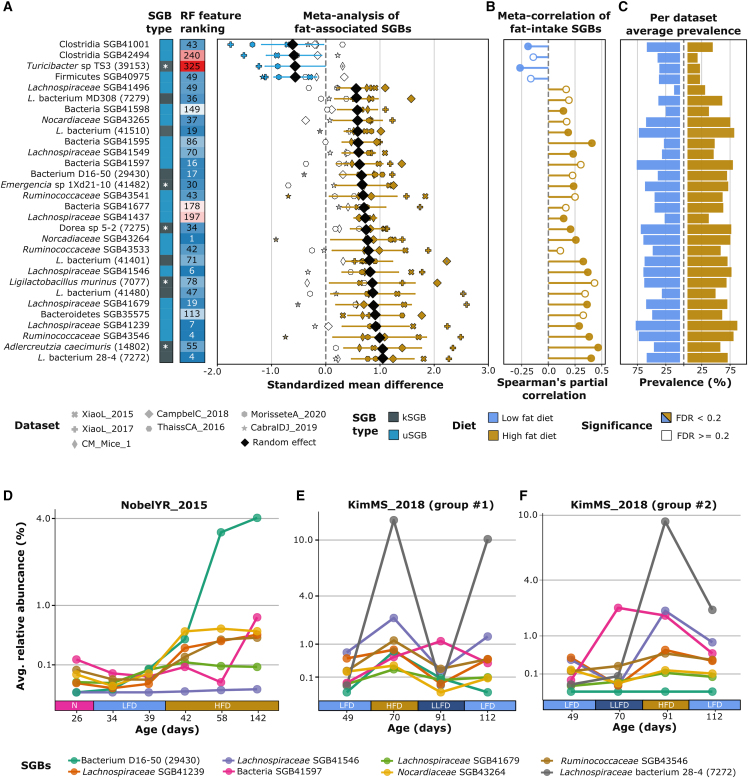
Meta-analysis of standardized mean differences of high- and low-fat-diet-related SGBs (A) Meta-analysis of standardized mean differences and a random-effect model, and random forest (RF) LODO average feature ranking. The 30 SGBs with the highest coefficients, FDR of the pooled effect size <0.2, and an average prevalence >20% are shown. Markers identify the single datasets, and the black diamonds indicate the random-effect coefficients. White symbols refer to FDR > 0.2, dark-yellow and light-blue symbols refer to a high-fat-related SGB and a low-fat-related one, respectively, both with an FDR < 0.2. Standardized mean differences have been extracted by the t score from a linear model, controlling by sex, age, genetic background, antibiotics usage, and sampling body-site. SGB abundances have been arcsin-square-rooted before the linear modeling. Species available in the RefSeq database are labeled with a white asterisk. *L*. denotes *Lachnospiraceae*. Horizontal colored (blue and dark-yellow) lines mark the 95% confidence interval for the pooled effect size. (B) Random-effect coefficients of a partial-correlation meta-analysis on diet-fat percentage, controlling also by duration of the diet, for the 30 SGBs most associated in the previous analysis. Correlations have been Fisher-*Z* transformed before the meta-analysis and then reverted back. SGB abundances have been arcsin-square-rooted before the linear modeling. (C) Per-dataset average prevalences (%) stratified by high-fat and low-fat mice. (D–F) Temporal fat-related trajectories for three mice from the Nobel et al.^7^ cohort and six mice from the Kim and Bae^48^ cohort. The lines are relative to the six top SGBs of the RF feature ranking chosen among the 30 strongest effect sizes in the meta-analysis, and describe the relative abundance (in square-root scale) of the single SGB in multiple dietary shifts. N, nursing; LFD, ∼10%(low)-fat diet; HFD, ∼40%(high)-fat diet; LLFD, 6.2%(low)-fat diet. (D) Trajectories in the Nobel et al.^7^ cohort, (E) trajectories in the Kim and Bae^48^ cohort, group #1, and (F) trajectories in the Kim and Bae^48^ cohort, group #2.

For each SGB and on each dataset separately, we then performed differential abundance analysis in high- vs. low-fat-diet regimes using linear models adjusted for sex, age, antibiotic treatment, mouse strain, and sampling body-site. The results from the linear models for each SGB were then pooled together in meta-analyses by a random-effects models^41^^,^^42^ (see STAR Methods). We identified 37 SGBs significantly associated with a high-fat diet and 10 SGBs with a low-fat diet (false discovery rate [FDR] < 0.2, considering SGBs at >20% prevalence and present in at least five datasets; Table S5). Importantly, 32 of these 47 biomarkers are uSGBs (chi-squared p on the expected even frequency: 0.01). Considering the 30 SGBs with the highest differences between the groups (Figure 2A), only 11 are kSGBs, 9 of which still belong to poorly characterized microbial species (i.e., *Turicibacter* sp. TS3, *Lachnospiraceae* bacterium MD308, bacterium D16-50, *Emergencia* sp. 1XD21-10, *Dorea* sp. 5-2, three distinct SGBs labeled “*Lachnospiraceae* bacterium,” and *Lachnospiraceae* bacterium 28-4; Figure 2A). The three SGBs with the largest effect sizes were *Lachnospiraceae* bacterium 28-4 (SGB7272), *Adlercreutzia caecimuris* (SGB14802), and *Ruminococcaceae* SGB43546 (aSMD = 1.05, 95% CI [0.48, 1.63], 1.05, 95% CI [0.33, 1.76], and 0.99, 95% CI [0.13, 1.86]; Q = 0.007, 0.04, and 0.13, respectively) corresponding to 4.0, 6.6, and 3.7 average fold abundance increases (see STAR Methods). Of note, these SGBs (one uSGB and two kSGBs that are, however, represented by poorly characterized genomes) belong to genera that have been recognized to convert primary to secondary bile acids^43^ or are described for their defensive role against intestinal high-fat-related inflammation.^44^ *A*. *caecimuris* (SGB14802) could in addition be potentially linked to the leptin increase in high-fat regimen, as well as correlated with serum primary bile acids.^45^^,^^46^^,^^47^

Only ten SGBs were increased in the low-fat group in at least five datasets, and the one showing the strongest association was *Clostridia* SGB41001 (a uSGB, aSMD = −0.61, 95% CI [−1.14, −0.08], Q = 0.13, fold change = 1.6, 95% CI [0.95, 2.7]). The sole SGB for which the genus had been previously identified among the low-fat-associated entries was *Turicibacter* sp. TS3 (SGB39153) (aSMD = −0.57, 95% CI [−1.08, −0.06], Q = 0.15, fold change = 4.5, 95% CI [1.0, 19.4]). This finding is concordant with previous studies that demonstrated *Turicibacter* declining in abundance in obesity,^49^ a negative correlation with the nuclear factor-κB-protein-complex^50^ that has a central role in inflammation signaling, and a lower abundance in patients with inflammatory bowel disease.^51^

Overall, the majority of the taxa associated with high- or low-fat diet were uSGBs, demonstrating that a metagenomic taxonomic profiling integrating both kSGBs and uSGBs (MetaPhlAn 4) is fundamental to overcoming the complexity of the mouse gut metagenome.

### Dietary fat intake correlates with microbial shifts

We next tested whether different percentages of dietary fat directly correlate with the abundance of each SGB, as the amount of fat intake in different studies can vary (Tables S1 and S2; Figure S1A). We thus performed a meta-analysis of partial Spearman’s correlations between the percentage of fat and the abundance of SGBs by fitting the regression model for the same available set of covariates considered in the previous meta-analysis as well as for the duration of the administration (Figures S3 and S5; Table S6). In total we found 44 SGBs correlating with the amount of fat (Spearman’s partial correlation Q < 0.2, detection in at least four studies), 32 of them (73%) being positively correlated (Figures S3B and 2B). As expected, we found a strong agreement between these significant correlations and the previous binary outcome association meta-analysis (Pearson’s, Spearman’s, and Kendall’s rho between the effect sizes of the two analyses = 0.79, 0.76, and 0.36, respectively; Pearson’s p = 1 × 10^−61^, Spearman’s p = 3 × 10^−53^, and Kendall’s p = 9.5 × 10^−19^; Figure S3C).

As in the binary association meta-analysis, the majority of the SGBs positively and negatively correlating with the amount of fat in diet were uSGBs (59% and 83%, respectively; altogether, 29 out of 44 hits were uSGBs, chi-squared p = 0.03), as well as the majority of SGBs correlating with the duration of the diet (19 out of 23 in total detected in a minimum of three studies, chi-squared p = 0.002). All of these uSGBs were not even assigned to any known genus, and many of them belong to the *Lachnospiraceae* and *Ruminococcaceae* families (9 and 4 out of 29 uSGBs, respectively). Overall, we detected more significant correlations with uSGBs than with kSGBs, and considering the top 15 SGBs per SGB type (uSGBs and kSGBs) correlating with the amount of fat, correlations with uSGBs were, by Fisher-*Z* transformation-based average, higher than with kSGBs (average Spearman’s rho = 0.30 vs. 0.29; see STAR Methods). These results suggest that uSGBs are more predominantly involved in response to a fat diet or to its duration than the kSGBs.

### Independent longitudinal datasets show consistent diet-related uSGB dynamics

To further validate our first set of observations, we analyzed two additional longitudinal datasets^7^^,^^48^ in which groups of mice were subjected to multiple dietary interventions. From the dataset by Nobel et al.^7^ we retrieved 18 metagenomes sampled from three mice (six time points each; Figure 2D), while the dataset by Kim and Bae^48^ is internally subdivided into two different groups of three mice each (Figures 2E and 2F) undergoing different multiple dietary treatments.

Out of the 30 SGBs having the highest discrimination coefficients in the cross-sectional meta-analysis (Figure 2A), we selected the eight uSGBs most highly ranked by the random forest algorithm (Table S7). Assessing the temporal trajectory of these uSGBs, we observed a clear, definite increase of their relative abundances in response to a higher fat intake in all three datasets (Figures 2D–2F; Wilcoxon signed-rank test p = 5.3 × 10^−5^, 5.9 × 10^−6^, and 0.0003, respectively, “(L)LFD” to “HFD”). The abundances decreased again when switching toward a lower fat intake, with changes observable within the considered daily time span (Figures 2E and 2F; Wilcoxon signed-rank test p = 1.3 × 10^−5^ and 0.0004, respectively, “HFD” to “(L)LFD”). Interestingly, the changes were directly observable also as a response to mild fat increase or decrease, with both groups of mice from Kim and Bae^48^ showing a minor decrease in the median abundance of the eight uSGBs when passing from a low-fat diet with a 10% fat intake (“LFD”) to a low-fat diet characterized by a 6.2% fat intake (“LLFD”; average relative abundance 0.87, 95% CI [0.73, 1.0], vs. 0.27, 95% CI [0.09, 0.45]; Wilcoxon signed-rank test, p = 0.056; Figures 2E and 2F). The strongest, uncharacterized cross-sectional microbial biomarkers of high-fat diet thus confirmed their link with diet in two interventional cohorts.

## Discussion

Here, we taxonomically profiled the gut microbiome of laboratory mice at an enhanced resolution by the integration of a massive number of MAGs (22,718 mice-derived MAGs) in the reference database of the MetaPhlAn 4 marker-based approach. To investigate whether this increased resolution can lead to the discovery of relevant associations between the mouse microbiome and host conditions, we focused on the analysis of the microbiome links with diet. We collected, manually curated, and profiled a set of nine mouse microbiome datasets (one of which was made available by this work), all characterized by the presence of multiple diet regimes differing in the percentage of fat. Machine learning and meta-analyses on the cohorts profiled with the MAG-enhanced database revealed cross-cohort associations with diet that are stronger than what was previously available and that were mostly driven by uncharacterized microbial species (uSGBs).

Our results highlight the need for inclusions of genomes from uncultured microorganisms in the process of taxonomic profiling of mice microbiome data and the key role that species available only through metagenomic analyses may play for host-microbiome interaction specifically in laboratory mice. Importantly, we showed that MetaPhlAn 4 is able to efficiently integrate uSGB profiling in the metagenomic analysis and thus largely improve the analysis of microbiome in mouse models.

### Limitations of the study

Our study and tools can be the basis for more nuanced study of nutritional effects on the microbiome and host-microbiome interactions in preclinical models. Improved study designs could, for example, account for the differences in saturated vs. polyunsaturated fat intake.^52^ In our study, we could not correct our analysis by the weight of the mouse at baseline or consider the polysaccharide nutritional content,^13^ although these aspects were shown to be only minor confounders with respect to the diet-induced obesity development.^53^^,^^54^^,^^55^ As the diet-microbiome-host links remain intricate and mouse models can be useful in studying them, it will also be crucial to extend the ability to profile uncharacterized aspects of the microbiome to microbial transcripts, metabolites, and proteins, and thus future work should be focused on integrative computational methods to profile microbiome mouse models with meta-omic approaches.^56^

## STAR★Methods

### Key resources table

**Table:** 

REAGENT or RESOURCE	SOURCE	IDENTIFIER
**Biological samples**		

Stool samples from CM_mice_1 cohort	This paper	NA

**Critical commercial assays**		

allPrep DNA/RNA Mini Kit	Qiagen, Hilden, Germany	Catalog No. 80284
Nextera XT DNA Library Preparation Kit	Illumina, California, USA	FC-131-1096

**Deposited data**		

Raw sequencing data (CM_mice_1 cohort)	This paper	NCBI-SRA BioProject: PRJEB52043

**Software and algorithms**		

MetaPhlAn (version 3.0.13)	^57^	https://github.com/biobakery/MetaPhlAn/
MetaPhlAn (version 4.0.0)	^30^	https://github.com/biobakery/MetaPhlAn/
metAML	^33^	https://github.com/segatalab/metaml

**Other**		

SILVA database	^58^	https://www.arb-silva.de/
RefSeq database	^40^	https://www.ncbi.nlm.nih.gov/refseq/

### Resource availability

#### Lead contact

Further information and requests for resources and reagents should be directed to and will be fulfilled by the lead contact, Nicola Segata (nicola.segata@unitn.it).

#### Materials availability

This study did not generate new unique reagents.

### Experimental model and subject details

The CM_mice_1 experiments were performed in the specific pathogen-free animal facility at IERP (INRAe, Jouy-en-Josas, agreement C78-720), in a temperature-controlled environment and with a strict 12h light/dark cycle. Animal experiments were performed according to the local ethical panel and the Ministère de l’Education Nationale, de l’Enseignement Supérieur et de la Recherche, France under agreement Apafis 19750-2019041014309428. Fifty females C57BL/6J from Janvier (France) were left for a minimum of 7 days acclimating (3–5 mice per cage/group). The gut microbiome of each group was then perturbed with one of the following challenges: vancomycin, colistin, penicillin, colistin + ofloxacin, vancomycin + penicillin, colistin + metronidazole, high fat diet (Envigo TD.88137; 42% fat), high milk fat diet (Envigo TD.97222, 38% fat), low tryptophan diet (Ssniff S9868-E020, 17% fat), high tryptophan diet (Ssniff S9868-E030, 17% fat), and control groups for each type of challenge (Envigo TD.97222 for high fat diet, Ssniff S9868-E010 for low and high tryptophan diet). Antibiotics treated mice were fed a conventional chow diet (Envigo TD.120508). Antibiotics were given in drinking water for 7 days at the following dosage: vancomycin, 0.5 g/L; metronidazole, 1 g/L; colistin, 1 g/L; penicillin, 1 g/L; ofloxacin, 0.25 g/L. Dietary intervention were maintained for 5 weeks before sacrifice (Table S2). At the end of the perturbation, mice were euthanized (70 days old), dissected and coecum and ileum aliquots were withdrawn and stored at −80°C.

### Method details

#### Public dataset collection and curation

We downloaded from NCBI a total of 15 public shotgun metagenomic datasets derived from fecal pellets, coecum or ileum content of lab-mouse metagenomes (N = 623 samples). Further criteria were the following variables to be available directly in the publication description of the cohort or by retrieval via the corresponding NCBI entry: age of the mouse, genetic background, antibiotics usage, sampling body-site, percentage of fat-intake and duration in days of the administration. Samples from animals who received fecal microbiota transplantation (FMT) from humans or other animals were excluded *a-priori*. Datasets involving diet regimen with a single amount of fat and datasets smaller than 20 samples were also excluded. The high-fat and low-fat labels used here for the diets were assigned by the authors of the original works in all cases but the dataset from Campbell et al.,^59^ in which this assignment was operated by the authors according to the presence of two groups of mice, one fed with a 13% fat-intake chow, the other with a 16.3%. The final number of datasets included was 8 (N = 525 samples). The final number of publicly available reads considered was 25.6 billions reads (avg. per sample = 48.7 Mln. 95% CI [9.7, 87.8]). For a summary of the datasets used in this study and the relative diet fat-percentages see Table S1. In total our dataset of publicly available samples consisted of 243 high-fat mouse samples, and 282 low-fat for which detailed information is available at Table S2.

#### Sequencing of microbiomes from mice undergoing several dietary fat-intakes

DNA was extracted using the allPrep DNA/RNA Mini Kit (Qiagen) following the manufacturer’s instructions. Sequencing libraries were prepared using the Nextera XT DNA Library Preparation Kit (Illumina), following the manufacturer’s guidelines. Sequencing was performed on a HiSeq2500 (Illumina) at the sequencing facility at University of Trento, Italy. Reads quality filtering was performed using trim_galore (parameters: --nextera --stringency 5 --length 75 --quality 20 --max_n 2 --trim-n), discarding all reads of quality less than 20 and shorter than 75 nucleotides. Filtered reads were then assigned to the C57BL/6J laboratory mouse genome (GCA_000001635.8), the PhiX genome and the SILVA database^58^ (ver. 132) for removal of host, contaminant and 16S material. Ninety-seven samples were obtained (26 classified as high-fat diet and 71 classified as normal chow), and 3.6 Bln. reads were produced (avg. per sample = 37 Mln. 95% CI [2.8, 70]).

#### Species- and SGB-level metagenomic taxonomic profiling

The selected 622 mice gut metagenomic samples were taxonomically profiled using MetaPhlAn 3^57^ (version 3.13; default parameters) and MetaPhlAn 4^30^ (version 4.0.0; default parameters). MetaPhlAn 4 relies on a markers database incorporating the Species-level Genome Bins (SGB) system to group both reference and metagenomic-assembled genomes into known (kSGBs) and unknown species (uSGBs).^31^ The updated database incorporates more than 1M microbial genomes, including 22,718 MAGs reconstructed from 1,906 mice gut metagenomes and spanning 540 mice-associated uSGBs (Table S3). RefSeq-based SGBs were selected using only SGBs spanned by reference genomes present in the RefSeq assembly database^40^ (accessed 9th February 2023).

### Quantification and statistical analysis

#### Statistical and machine-learning based approaches

Group statistical differences were assessed via two-tailed Wilcoxon-signed rank test and two-tailed binomial test (using Scipy python library, ver. 1.4.1). Beta-diversity analysis was based on Bray-Curtis pairwise distances and computed on the SGB-relative abundances using Scipy (ver. 1.4.1) and Scikit-Bio (ver. 0.5.6). Significance of the pairwise-distances matrices were assessed using univariate Permanova analysis in python (Scikit-Bio library, ver. 0.5.6). Variable importance (adjusted R^2^) was estimated using the *capscale* function from the vegan package (ver. 2.5.7.)^60^ (multivariate Permanova) on Bray-Curtis dissimilarity matrices by a model of the type: “*beta-diversity ∼ study-id* + *age of the mouse in days* + *mouse sex* + *sampling body-site* + *dietary regimen*”. We run the function *ordistep* (vegan, ver. 2.5.7)^61^ which estimates the best model by covariate importance on the output of *capscale*. Shannon-diversity, Gini-Simpson-diversity, and sample richness were computed with custom python scripts. Intervals at 95% confidence-level were computed as 1.96 times the standard error of the estimated mean for standardised mean differences in alpha and beta-diversity and for SGB-count averages. Average proportions of uSGB versus kSGB and other relative abundances were considered binomially distributed and confidence intervals were computed accordingly. Machine-learning experiments were run using the scikit-learn random forest (RF^32^) classifier implementation (ver. 0.24.2) hosted in the metAML software.^33^^,^^36^ In particular, we set up RF with 1,000 estimator trees, 5 maximum number of samples per each leaf, no-fixed-maximum depth for each tree, the square-root of the feature-space length as input to each tree, and Shannon-entropy as impurity criterion. Shannon-entropy was chosen according to Thomas et al.,^36^ the number of trees was chosen according to Behini et al.,^57^ while all the other parameters are set with their default values. Baseline mice (in the case of multiple timepoints) present in a dataset storing at least one control and one high-fat diet sample and classified as having received a high-fat diet were considered the positive class; their counterpart not under high-fat diet was considered the control class. The relative abundances of the SGBs were used as features after transformation with the arcsin square root. Scoring index was the area under the receiver operating characteristic curve (AUC). Several performance assessment techniques were adopted. Cross Validations (CV) were carried out on single datasets using 10-fold, balanced-by-class splits and 10 random repetitions. For leave-one-dataset-out (LODO) each cohort was iteratively used as the testing set while the algorithm was trained on all the other cohorts. The other type of test consists in training an algorithm on a single cohort and testing it on a different one. Each experiment was repeated 10 times. Final AUC values were thus averages of 100 tests in CVs and of 10 in transfers and LODOs. AUCs were considered asymptotically normal and their confidence intervals were based on the t-distribution with n-1 degrees of freedom where n is the number of datasets considered, 6, times the number of randomizations of the experiment, 20. RF average feature ranking was extracted averaging over the rank of each LODO test and computed only from the training sets to avoid overfitting.

#### Standardised mean differences meta-analysis

For each SGB, we evaluate the per-dataset dependency of the single feature to the contrast high-fat/low-fat using linear models. For each dataset and each SGB, a linear model was evaluated of the type: *“feature ∼ age* (*days*) + *sampling-body-site* + *mouse-background* + *antibiotic-usage* + *diet-type”*. Sampling body-site, genetic background, antibiotic-usage and diet-type (high-fat/low-fat) were encoded as categorical variables. From each of these models, a standardised effect-size and its standard errors were computed as a measure equivalent to the Cohen’s d as described in Nakagawa and Cuthill,^62^ starting from the t-value of the relative covariate. Significance of the diet-feature relationship was assessed with the Wald-test. Linear models, t-tests and their significance were computed using ordinary least squares (OLS) in Statsmodels python library (Seabold and Perktold^63^, ver. 0.11.1). Effect-sizes were then combined in random-effect meta-analysis using a python script implementing the procedure described in Borenstein et al.^64^ and the Paule-Mandel heterogeneity from Statsmodels (ver. 0.11.1). Meta-analysis coefficients (average Standardised Mean Differences) confidence intervals at 95% confidence were computed as described in Borenstein et al.^64^ Wald-Ps and Spearman’s Ps from the single datasets in the first and in the second meta-analyses, as well as random-effect raw p-values were then corrected for false discovery rate (FDR) using Benjamini-Yakuteli procedure (Scikit-Bio, ver. 0.5.6) and a significance value of 0.2.

#### Log fold changes meta-analysis

An epsilon was added to the zero values (0.0001) of the SGB relative abundances. Those were then log-2 transformed and averaged, and the difference was taken (log fold change). The first element of the log ratio was chosen as the high-fat group when the standardised mean difference effect-size was positive and as the low-fat group when it was true the viceversa. Variances of the log fold changes were computed as the sum of the variances of the two log variates being independent in all cases. Log fold changes were summarised in a meta-analysis by fixed effect-model using these variances and these effects. The same script as before was used in this step. The standard error of the log fold changes meta-analysis coefficient was taken as the square root of the variance of the averaged effect divided by the square root of the number of datasets analyzed, and 95% confidence intervals were computed as the average effect ± this standard error multiplied by the 97.5 quantile of a t distribution with number of datasets minus 1 degrees of freedom. Results in percentage were then computed as 2 elevated to mean and confidence intervals.

#### Correlation meta-analyses

Two similar procedures were carried out meta-analysing Spearman’s partial correlation coefficients which had been computed, together with their significances, using the Pingouin python library^65^ (ver. 0.3.7). Correlation coefficients were Fisher-Z transformed, summarised and reverted back using a custom python script reproducing the result from Balduzzi et al.^66^ We meta-analysed both the duration of the diet-administration correcting by the whole set of covariates and the percentage of fat, and the fat-percentage controlling by the whole set of covariates plus the duration of the administration. Significance of the random-effect coefficients were computed as described in Borenstein et al.^64^ Wald-Ps and Spearman’s Ps from the single datasets in the first and in the second meta-analyses, as well as random-effect raw p-values were then corrected for False Discovery Rate (FDR) using Benjamini-Yakuteli procedure (Scikit-Bio, ver. 0.5.6) and a significance value of 0.2. Spearman’s correlations were averaged after being Fisher-Z transformed and the reverse Fisher-Z transformation was applied on the resulting value. Validation on the temporal trajectories on the two validation datasets^7^^,^^48^ were carried out on SGB relative abundances. Samples undertaking antibiotics and dams from Nobel et al.^7^ were excluded.

## Data Availability

• The 97 mouse metagenomes produced in this study are publicly available at the European Nucleotide Archive under accession number PRJEB52043. Public cohorts accession codes are available in Table S1, while manually curated metadata for private and the public cohorts are available in Table S2. (NCBI) with accession numbers GenBank:CP091140-CP091142 and CP091091. • This paper does not report original code. • Any additional information required to reanalyze the data reported in this work is available from the lead contact upon request.

## Supplemental information

Document S1: Figures S1–S3

Table S1. The nine datasets considered in this study, related to STAR Methods

Table S2. Curated sample metadata for 622 samples with sequencing statistics, related to STAR Methods

Table S3. Mouse-derived MAGs included in the catalog, related to STAR Methods

Table S4. Permanova analysis of covariates on microbiome composition (MetaPhlAn 4), related to Figure 1

Table S5. SGB high-fat/low-fat meta-analysis, related to Figure 2

Table S6. SGB fat-% Spearman’s correlation meta-analysis, related to Figure 2

Table S7. Random forest feature rankings, related to Figure 2

Document S2. Article plus supplemental information
