# Supplementary material for: MetaPhlAn 4 profiling of unknown species-level genome bins improves the characterization of diet-associated microbiome changes in mice
Source: Cell Rep. 2023 May 3;42(5):112464. doi: 10.1016/j.celrep.2023.112464 (PMC10242440; doi:10.1016/j.celrep.2023.112464)
Supplement: Document S1: Figures S1–S3 [file mmc1.pdf]

Cell Reports, Volume *42*

Supplemental information

MetaPhlAn 4 profiling of unknown species-level genome bins improves the characterization of diet-associated microbiome changes in mice

Paolo Manghi, Aitor Blanco-Míguez, Serena Manara, Amir NabiNejad, Fabio Cumbo, Francesco Beghini, Federica Armanini, Davide Golzato, Kun D. Huang, Andrew M. Thomas, Gianmarco Piccinno, Michal Punčochář, Moreno Zolfo, Till R. Lesker, Marius Bredon, Julien Planchais, Jeremy Glodt, Mireia Valles-Colomer, Omry Koren, Edoardo Pasolli, Francesco Asnicar, Till Strowig, Harry Sokol, and Nicola Segata

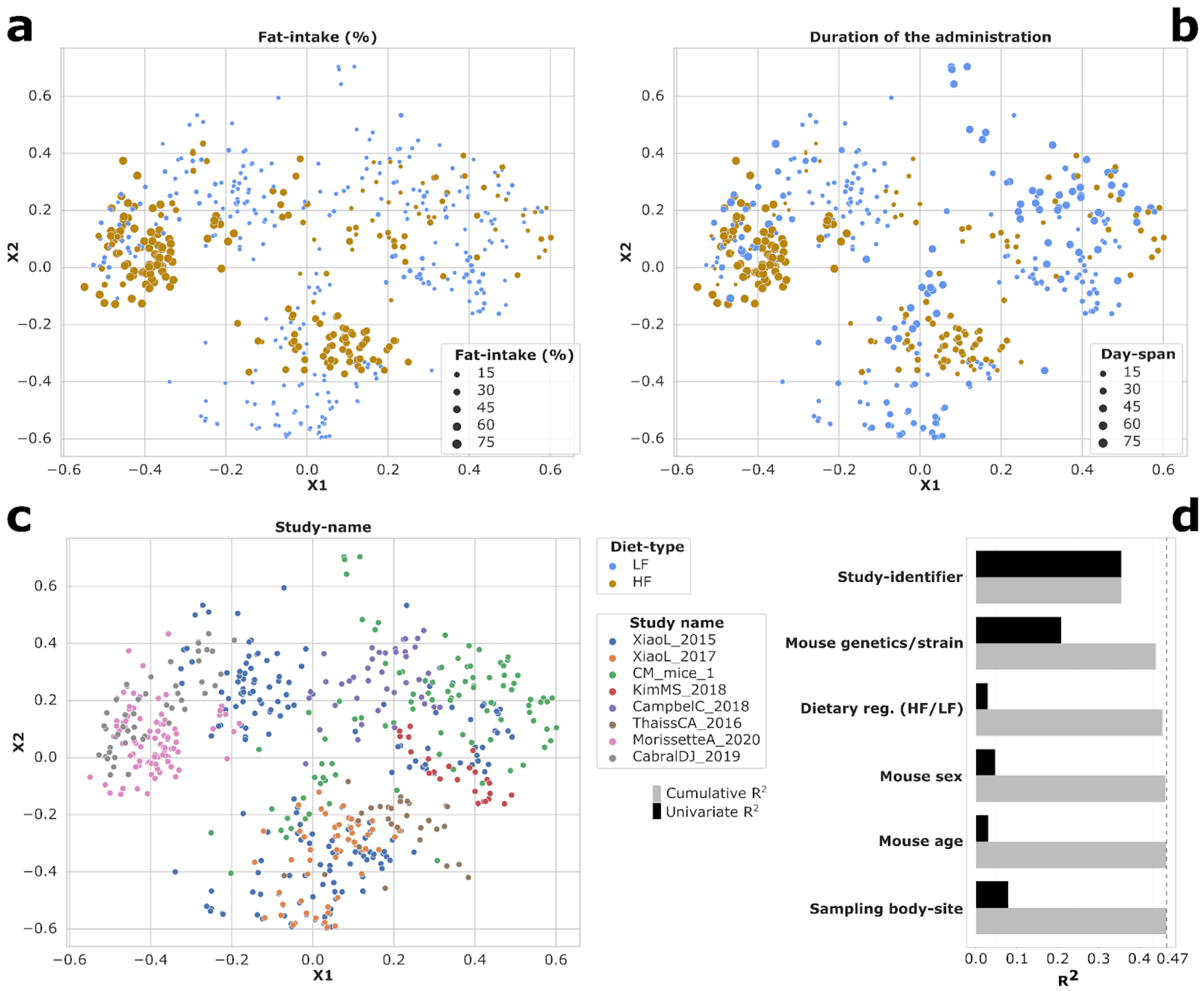

Figure S1: Bray-Curtis beta-diversity analysis ordination plots and multivariate Permanova analysis. Ordination plots representing the first 2 principal components of a Multi Dimensional Scaling (MDS) performed on Bray-Curtis pairwise dissimilarity matrices computed on arcsine-square-rooted Species-level Genome Bins (SGBs) relative abundances from MetaPhlAn 4 of a set of 9 mouse datasets. Dots are coloured according to their belonging to a high-fat dietary regimen or a low-fat dietary regimen in their study. Dots are sized according to the percentage of fat intake in the diet in **a)** and by the duration of high- or low-fat dietary administration in **b)**. **c)** same ordination plot with dots coloured based on the study name. CM_mice_1 refers to the dataset produced in this study. **d)** Univariate and cumulative coefficients of determination (adjusted R^2) resulting from a multivariate permanova model linking the beta-diversity Bray-Curtis pairwise distance matrix with study-identified, mouse genetics, mouse sex, mouse age, sampling body-site, and mouse dietary regimen (high-fat or low-fat) as fixed effects.

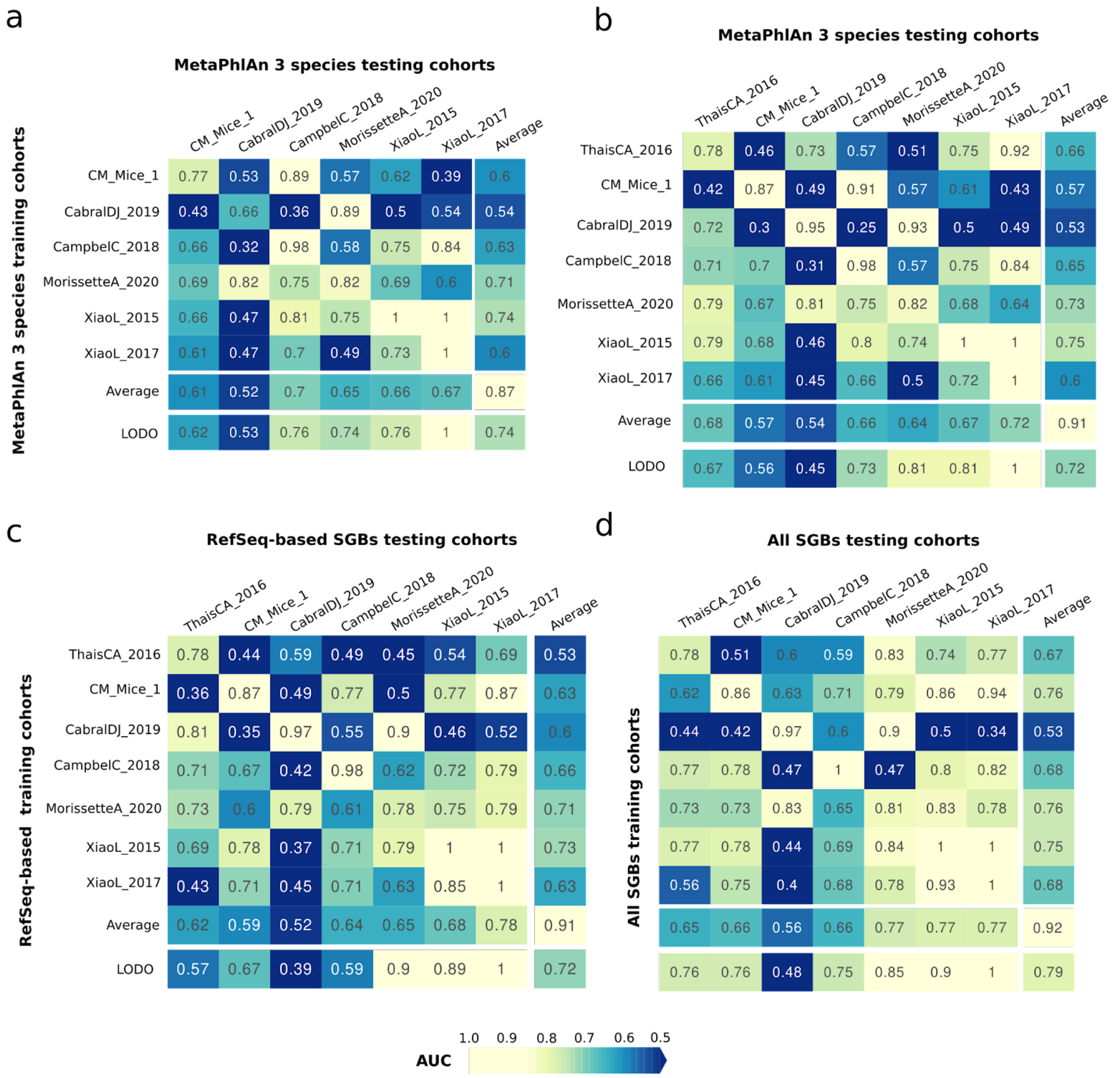

Figure S2: Cross-prediction matrices for the prediction of a high-fat vs. a low-fat diet using a Random Forest (RF) classifier trained on arcsine-square-rooted microbial relative abundances from MetaPhlAn 3 and MetaPhlAn 4. a-b) Cross-prediction matrices for the prediction of a high-fat vs. a low-fat diet using a Random Forest classifier trained on arcsine-square-rooted relative abundances computed via MetaPhlAn 3 from samples not undergoing antibiotics treatment only (**a**), and considering all samples (**b**). **c-d)** Cross-prediction matrices for the prediction of a high-fat vs. a low-fat diet using a Random Forest classifier trained on arcsine-square-rooted relative abundances of SGBs spanned by RefSeq reference genomes (**c**) or all the SGBs available in MetaPhlAn 4 from samples not undergoing antibiotics treatment only (**d**). Cells in the matrices represent AUCs obtained by the classifier trained on the corresponding row-dataset and tested on the corresponding column-dataset. Diagonal values are 10-fold cross-validations AUC values. The "LODO" row reports the Leave-One-Dataset-Out AUC values, obtained by training the algorithm on each cohort but one and testing it on the left-out cohort, performed iteratively on all the cohorts.

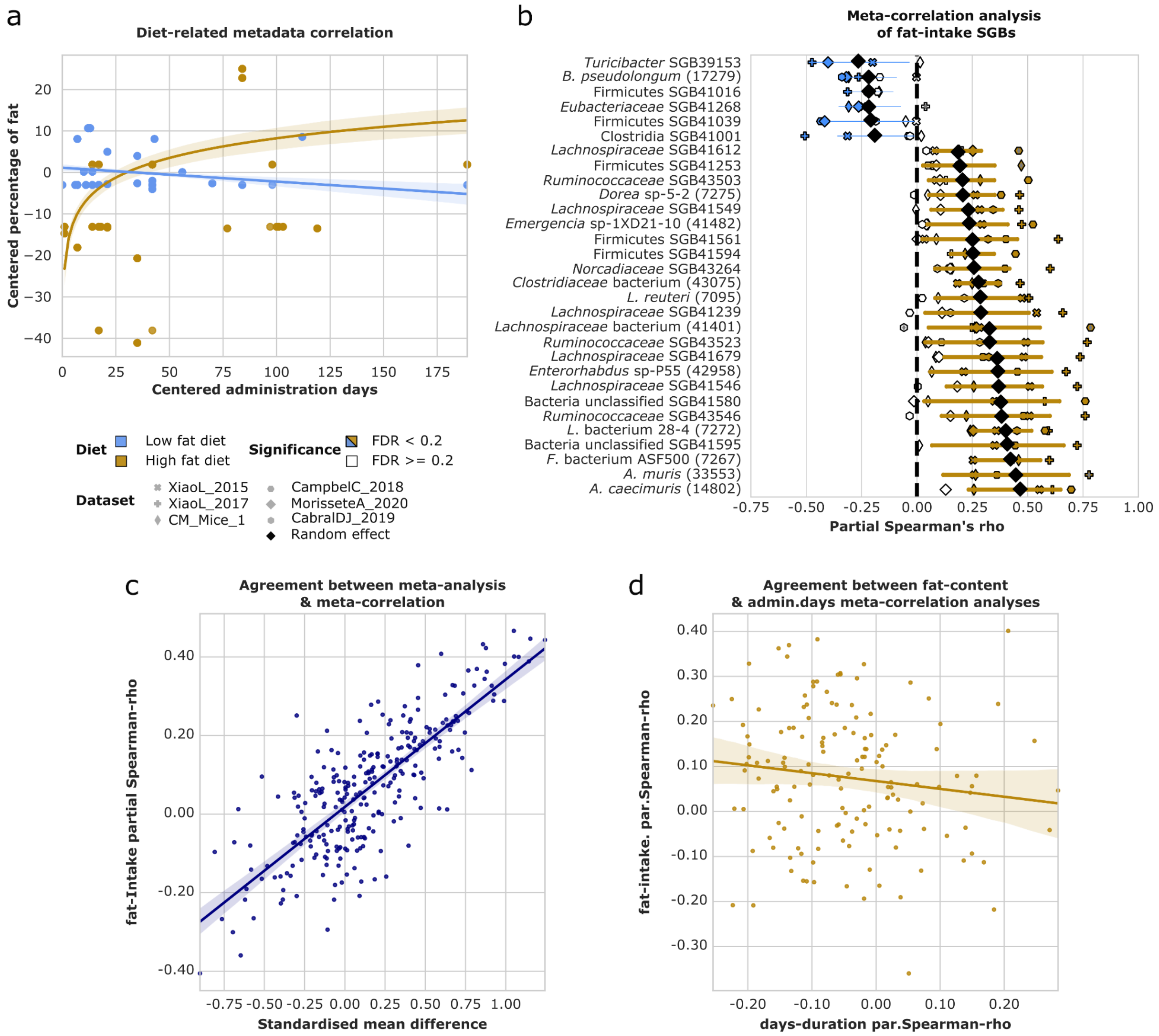

Figure S3: metadata intrinsic correlations & agreement between fat-intake and diet-duration partial correlation analyses. a) regression plots of the duration of the administration of the diets (x-axis) and the centered fat-percentage (y-axis). Samples are coloured depending on the high- or low-fat-classification of the diet. Y-axis has been log-scaled in the high-fat group. **b)** 30 SGBs showing the highest correlation with dietary fat-intake in a meta-analysis of Spearman's correlation coefficients partialized by by age of the mouse, antibiotic usage, sampling body-site, genetic background and duration of the diet administration, an average prevalence > 20%, a minimum presence in 4 datasets, and an FDR of the pooled effect-size < 0.2. Markers identify the single datasets, the black diamond indicates the random effect coefficient. White symbols refer to FDR > 0.2, dark-yellow and light-blue symbols refer to a high-fat-related SGB and a low-fat-related one, respectively, both with a FDR < 0.2 in the corresponding dataset. Horizontal lines (blue and dark-yellow) mark the 95% CI of the pooled effect-size. SGB abundances have been arcsin-square-rooted before the partial correlation. **c)** Scatterplot with meta-analysis coefficients from both analyses computed from SGBs present in a minimum of 4 studies are reported. **d)** scatterplot of the partial correlation meta-analyses of SGB arcsine-square-rooted abundances and fat-percentage in the diet (Spearman's rho, corrected by antibiotics usage, days of administration and dataset, x-axis), and SGB arcsine-square-rooted abundance and duration of the administration of the diet (Spearman's rho, corrected by antibiotics usage, fat-intake and dataset, y-axis).
